# Biochemical mechanisms underlying the differences in fruit characteristics among three kumquat varieties

**DOI:** 10.3389/fpls.2025.1640218

**Published:** 2025-09-05

**Authors:** Manti Li, Zili Yi, Haole Shang, Changwei Zhu, Hongmei Huang

**Affiliations:** ^1^ College of Bioscience and Biotechnology, Hunan Agricultural University, Changsha, China; ^2^ Hunan Engineering Laboratory of Miscanthus Ecological Applications, Hunan Agricultural University, Changsha, China

**Keywords:** traits, non-targeted metabolomics, flavor, sucrose, limonin, flavonoids, active ingredients

## Abstract

To elucidate different fruit trait mechanisms among Changshou (FOT) (*Fortunella obovata* Tanaka), Liuyang (FCSLY) (*Fortunella crassifolia* Swingle), and Huapi (FCSHP) (*Fortunella crassifolia* Swingle) kumquats, fruit traits, and their metabolite compositions were comprehensively analyzed. The main factors affecting fruit sweetness and bitterness are the sucrose and limonin contents. Differential metabolite analysis showed that FOT peels had the highest redness and more up-regulated anthocyanin and carotenoid compounds. Therefore, anthocyanin and carotenoid metabolites were related to the kumquat peel color. FOT peels had the highest flavonoid content. A total of 1719 metabolites were identified using non-targeted metabolomics, Flavonoid metabolites are more abundant in kumquat peels than in seeds, so the peel’s medicinal value is higher. The total limonin content in seeds is higher than in peels, so the seeds are used as raw materials for extracting limonin compounds. This study analyzed the biochemical mechanisms of fruit trait differences in three kumquat (*Fortunella* Swingle) varieties and provided a reference for targeted kumquat development and utilization.

## 1 Introduction

Kumquat originates from China and boasts a long history of cultivation. It can be classified into six species: *Fortunella hindsii* Swingle, *Fortunella polyandra* Tanaka, *Fortunella margarita* Swingle, *Fortunella japonica* Swingle, *Fortunella crassifolia* Swingle, and *Fortunella obovata* Tanaka (Palma and D’Aquino, 2018). Kumquat is closely related to citrus, and both belong to the citrus plants of Rutaceae. However, they exhibit distinct differences in their phytochemical compositions (Li et al., 2023; Ogawa et al., 2001) the peel, offering rich nutrients such as flavonoids, phenolic acids, carotenoids, vitamins, and polysaccharides (Liu et al., 2019). Due to its phytochemical profile, it has been traditionally used in Chinese herbal medicine (Liu et al., 2019). Its peel also contains abundant essential oils and lipids (Liu et al., 2019). Modern medical experiments have demonstrated kumquat’s efficacy in preventing vascular rupture, reducing capillary fragility, improving vascular permeability, and delaying vascular sclerosis. It also exhibits a two-way regulatory effect on blood pressure and enhances the body’s cold resistance, thus helping prevent colds (Abirami et al., 2014; Tan et al., 2016).

Current metabolomic studies on kumquat remain limited compared with Citrus fruits, particularly regarding flavonoids, coumarins, phenolic acids and essential oils. (Min-Hung et al., 2017) revealed that traditional small citrus fruits in Taiwan (kumquats and calamondins) predominantly contain bioactive metabolites including terpenes in essential oils, flavonoids, phytosterols and limonoids, all of which contribute to their antioxidant properties. The juice of kumquat contains flavonoids as its primary metabolites (Barreca et al., 2011). Phloretin 3’5’-di-C-glucoside represents the most abundant flavonoid. Several other flavonoid components were identified including avicularin 3,6-di-C-glucoside which was reported for the first time in kumquat juice. Metabolic profiling and gene expression analysis revealed cultivar-specific differences between ‘Huapi’ kumquat (*F.crassifolia* Swingle) and its wild type. The peel of ‘Huapi’ kumquat contained higher levels of hexosylated flavonoids compared to the wild type. In contrast, its pulp showed lower flavonoid content. The differentially accumulated lipids were mainly lysophosphatidylcholines and lysophosphatidylethanolamines. These lipids were less abundant in both peel and pulp of ‘Huapi’ kumquat (Ma et al., 2021). Sadek et al (Sadek et al., 2009) investigated the peels of two Fortunella margarita specimens from Greece and Egypt. They identified major metabolites including phenolic acids and derivatives. Flavanones were detected in both C-glycosyl and O-glycosylated forms, along with flavonols and chalcones. The ethyl acetate fractions showed high polyphenol content and strong antioxidant activity. However, these studies primarily focused on the metabolite composition, content variations and bioactivities of kumquat. No comparative metabolomic analysis was conducted between kumquats and citrus fruits, and the metabolomics of kumquat seeds remains completely unexplored. Furthermore, the metabolome of ‘Changshou’ kumquat is still lacking.

‘Changshou’ kumquat is a hybrid between kumquat and Citrus species. Kiichi Yasuda’s CMA analysis revealed E-type Citrus chromosomes in Changshou kumquat (Yasuda et al., 2016). This confirms its hybrid origin from both genera. ‘Changshou’ kumquat exhibits distinct phenotypic differences from traditional Fortunella crassifolia. Its peel shows easier detachment characteristics. This feature facilitates peel processing and component extraction. Both ‘Liuyang’ kumquat and ‘Changshou’ kumquat possess prominent oil glands. In contrast, ‘Huapi’ kumquat exhibits smooth peel with pale coloration, sparse oil gland density, and reduced pungency. Current knowledge gaps remain in Fortunella metabolomics, particularly for ‘Changshou’ kumquat. This study presents the first comparative metabolomic analysis of peel and seed tissues from ‘Changshou’ kumquat, ‘Liuyang’ kumquat, and ‘Huapi’ kumquat, while also incorporating the fruit traits of these three kumquat varieties. The results reveal metabolic bases underlying their phenotypic differences. These findings facilitate understanding metabolite variations between kumquat and Citrus. This work provides fundamental data for identifying unique metabolites and elucidating functional differences between these medicinal-edible plants.

## 2 Materials and methods

### 2.1 Plant materials

The study investigated two kumquat species. *F. obovata* Tanaka (commonly called ‘Changshou’ kumquat, voucher numbers: IMC0088085, deposited at the Chinese Virtual Herbarium) is a small spineless tree (Zhang, 2019). It bears dark green, broad-ovate leaves (4.5 cm long × 2-3.5 cm wide). The petals are approximately 1.48 cm long × 0.15 cm wide. The mature fruits are pyriform and lemon-yellow (2.5–4 cm longitudinal × 2.5-3.5 cm transverse diameter), averaging 23.0 g in weight. *F. crassifolia* Swingle (voucher numbers: PE 01470066, deposited at the Chinese Virtual Herbarium) is a shrub-like small tree with nearly spineless branches (Zhang, 2019). It features winged leaves that are ovate, slightly thick, and acuminate at the apex (4.4-7.5 cm long × 2.2-3.4 cm wide). The flowers are relatively large, with a spreading diameter of 1.9-2.0 cm. Petals measure 0.7-0.9 cm long × 0.3-0.4 cm wide. As the primary commercial cultivar in kumquat, its fruits are oblong-ovate (2.3-3.1 cm transverse diameter × 2.3-3.3 cm longitudinal diameter), averaging 12.7-20.6 g in weight. The edible fruits contain almost no oil glands and produce polyembryonic seeds. The study focused on two horticultural cultivars derived from *F. crassifolia* Swingle: ‘Liuyang’ kumquat and ‘Huapi’ kumquat. “Liuyang” kumquat is the main variety and has a long cultivation history in Liuyang City, Hunan Province, China. The ‘Huapi’ kumquat, identified through screening efforts in the 1980s, represents a naturally occurring seedling mutant derived from ‘Rongan’ kumqua (*F. crassifolia* Swingle).

The experimental materials included peel and seed samples from three kumquat cultivars:(1) ‘Changshou’ kumquat (*F. obovata* Tanaka; full name: FOT, peel: FOTP, seed: FOTS), (2) ‘Liuyang’ kumquat (*F. crassifolia* Swingle; full name: FCSLY, peel: FCSLYP, seed: FCSLYS), and (3) ‘Huapi’ kumquat (*F. crassifolia* Swingle; full name: FCSHP, peel: FCSHPP, seed: FCSHPS). The materials are currently cultivated at the experimental base of Hunan Agricultural University, located in Guandu Town, Liuyang City (28°347622 N; 113.886829 E). The region experiences a subtropical monsoon humid climate, with an average temperature of 17.4°C, an annual average of 1650 hours of sunshine, and an average annual precipitation of 1640 millimeters. The soils are typical Quaternary red clay (Ultisols) developed from plate shale weathering, characteristic of subtropical citrus-growing regions in Central China. Soil analysis showed acidic conditions (pH 5.2 ± 0.4) with moderate fertility (organic matter 2.8 ± 0.5%, total N 1.3 ± 0.2 g/kg, available P 15.3 ± 3.2 mg/kg, exchangeable K 125 ± 18 mg/kg).

Under identical management conditions, fruits from three kumquat cultivars were collected from the same orchard, with mature fruits harvested from five randomly selected trees per cultivar. Five fruits of the same size were selected from different locations on each tree. The peels and seeds were separated, and the peels of the same variety were mixed. Similarly, the seeds of the same variety were mixed. The samples were immediately frozen in liquid nitrogen for approximately 15 minutes and divided into seven parts, which were labeled accordingly. Three parts were utilized to determine the total flavonoid content(TFC), total phenolic content(TPC), total limonoid content(TLC), and total fat content(TFAC) in kumquat. The remaining four parts were used for LC-MS metabolomics analysis.

### 2.2 Determination of color, flavor, and oil gland density in ripe fruit

Fruit color was measured using a colorimeter (Konica Minolta CR-400, Japan). Five fruits were selected for each variety, and the color difference values (L*, a*, b* values) were measured in three directions along the equator.

According to the sensory analysis method, the selection of tasters, sample selection and preparation, and error control during sensory evaluation were conducted. In accordance with the sensory analysis method (China National Institute Of Standardization, 2020), specifically the ‘A-non-A’ test method, 5–10 tasters were invited to taste the fruits of each variety, with each taster sampling five fruits from each variety.

The oil gland density was assessed following the citrus germplasm resource description specification and data standard (Dongjiang and Gong, 2006). During the fruit maturity stage, ten representative fruits of uniform size from the middle of the canopy were selected as observation samples. The number of oil glands along the equator of the peel was observed under a magnifying glass. Based on the oil gland count per unit area, the oil gland density level was determined for each variety. Oil gland densities below 45/cm^2^ were classified as sparse, densities between 45~65/cm^2^ were categorized as medium, and densities exceeding 65/cm^2^ were considered dense.

### 2.3 Determination of sugar and acid content in mature fruits of three kumquat varieties

The 20 kumquat fruits from each variety were sliced into pieces, freeze–dried, and ground into powder (Dongjiang and Gong, 2006). A total of 0.75 g of powder was mixed with ultra-pure water to create a suspension. Three samples were prepared for each variety. The suspension was then subjected to a 70°C water bath for 30 minutes, followed by supernatant collection. The supernatant was further diluted in a 25mL volumetric flask to obtain the loading solution for the quantification of sugar and acid content. The sugar analysis included sucrose(SC), glucose(GC), and fructose(FC), while the acid analysis comprised citric acid(CA) and malic acid(MA).

The sugar and acid contents were analyzed using high-performance liquid chromatography (HPLC)(LC-40, SHIMADZU Co., Ltd., Kyoto, Japan). For sugar determination, the chromatographic column employed was a Shodex SUGAR SP0810(8.0 mm × 300 mm, 7.0μm), operated at a temperature of 80°C. The mobile phase consisted of ultrapure water at a flow rate of 0.5 mL/min. The analysis was performed using a refractive index detector (RID 20A, SHIMADZU Co., Ltd., Kyoto, Japan), and the injection volume was set at 5 µL. As for acid determination, the chromatographic column utilized was an Inertsil ODS-2 C18 column (4.6 mm × 250 mm, 5 μm; GL Sciences, Inc., Japan). The solvent used was 50 mM KH_2_PO_4_ at a flow rate of 0.6 mL/min. The eluted peaks were detected using an ultraviolet detector (Shimadzu, Japan) operated at 210 nm, and each acid was quantified using the area under the peak relative to that of known standards. Triplicate tissue samples were also analyzed.

### 2.4 Determination of total flavonoids, total phenols, limonoids, and total lipids in peels and seeds from three kumquat varieties

The TFC was determined using the NaNO_2_-AlCl_3_-NaOH method from a biochemical kit (NMKD0120, Norminkoda Biotechnology Co., Ltd., Wuhan, China) (Hu et al., 2023). In each experiment, about 0.1 g of the flesh tissue was extracted using 2 mL of extraction solution in 60°C water in an ultrasonic bath for 30 min. The solution was centrifuged at 10,000g at 25°C for 10 min, and the supernatant was collected. The supernatant was combined and standardized to a final volume of 10 mL with extraction solution. The spectrophotometer microplate reader was preheated for 30 min, and the absorbance was measured at the wavelength of 510 nm. The concentration of total flavonoids was expressed as mg of rutin equivalent (RE) per gram fresh weight (FW). A rutin standard was used as the positive control. These analyses were performed in triplicate.

The TPC was extracted and quantified using a total phenol biochemicals kit (NMKD0120, Norminkoda Biotechnology Co., Ltd., Wuhan, China) (Sun et al., 2023). Under alkaline conditions, phenols reduce tungsten molybdate to form a slightly blueish compound with a characteristic absorption peak at 760 nm. About 0.1 g of tissue and 2.0 mL of the extract was added, and the TPC was extracted using an ultrasonic extraction. The ultrasonic power was 300 W, and the mixture was crushed for 5 s, intermittently for 8 s, before being extracted for 30 min at 60°C. The mixture was centrifuged for 10 min at 12,000 rpm, at 25°C, and the supernatant was taken and diluted to 2.5 mL with extract solution. After the extraction, the absorbance at 760 nm was determined using a spectrophotometer to obtain the TPC in each sample. A total of 1 mg of tannic acid was added to 1 ml of extractive solution and then diluted to different concentrations to plot the standard curve, and the results were expressed as mg of tannic acid equivalent (TAE) per gram fresh weight (FW). Tannic acid standard was used as the positive control. The analyses were performed in triplicate.

The total content of limonoids is determined by colorimetric method (Qingguo Tian, 1999) using the biochemical reagent kit (NM-W-0739, Norminkoda Biotechnology Co., Ltd. Wuhan, China). The experiment used petroleum ether for degreasing, followed by extraction with acetone and detection of absorbance at 470nm. Using limonin as a standard(y = 0.1095x - 0.0046, R2 = 0.9914), calculate the content in the sample. The results were expressed as mg of imonin equivalent per g fresh weight (mg/g FW). The analyses were performed in triplicate.

The TFAC was extracted using the Soxhlet extraction method (Aravind et al., 2021). Briefly, about 1g of tissue was dried and put into a Soxhlet extractor to extract TFAC with petroleum ether as the solvent and turned back 60 times at 65°C using a kit from Norminkoda Biotechnology Co., Ltd. Wuhan, China.

### 2.5 Extraction process for metabolite analysis

A total of 25 mg of the sample was weighed into an EP tube after grinding with liquid nitrogen, and 500 μL extract solution (methanol:water = 3:1, with an isotopically labeled internal standard mixture) was added. Then, the samples were homogenized at 35 Hz for 4 min and sonicated for 5 min in an ice–water bath. The homogenization and sonication cycle was repeated three times. Then, the samples were incubated for 1 h at −40°C and centrifuged at 12000 rpm (RCF=13800(×g), R= 8.6cm) for 15 min at 4°C. The resulting supernatant was transferred to a fresh glass vial for analysis.Besides, the quality control (QC) sample was formed by mixing an equal aliquot of the supernatants from all samples (Wu et al., 2018).

### 2.6 LC-MS/MS analysis

LC-MS/MS analyses were performed using a UHPLC system (Vanquish, Thermo Fisher Scientific) with a UPLC HSS T3 column (2.1 mm × 100 mm, 1.8μm) coupled to an Orbitrap Exploris 120 mass spectrometer (Orbitrap MS, Thermo). The mobile phase consisted of 5 mmol/L ammonium acetate and 5 mmol/L acetic acid in water (A) and acetonitrile (B). The gradient profile was as follows: gradient program, 0–0.7 min, 1% B, 0.35 ml/min; 0.7–11.8 min, 99% B, 0.35 ml/min; 11.8–14.6 min, 1% B, 0.50 ml/min; 14.6–15 min, 1% B, 0.35ml/min. The auto-sampler temperature was 4°C, and the injection volume was 2μL.

The QE HFX mass spectrometer was used for its ability to acquire MS/MS spectra on Information-Dependent Acquisition mode in the control of the acquisition software (Xcalibur, Thermo). In both positive and negative ion modes, the acquisition software continuously evaluates the full scan MS spectrum. The Electron Spray Ionization source conditions were set as follows: a sheath gas flow rate of 30 Arb, Aux gas flow rate of 25 Arb, a capillary temperature of 350°C, full MS resolution as 60000, MS/MS resolution as 7500, collision energy as 10/30/60 in Normalized Collision Energy mode, a spray voltage of 3.6 kV (positive) or −3.2 kV (negative), respectively. Comparative analysis of differential metabolites was conducted by integrating detection data from both positive and negative ion modes.

After the original data were converted intomzXML format using the software ProteoWizard (https://proteowizard.sourceforge.io/),the Rpackage XCMS (version 3.2) was used for the peak recognition, extraction, alignment, and integration. The preprocesses of the original data included the following: (1) Data filtering: The filtering standard was to remove the data with no definite substance name or no spectrum comparison similarity. (2) Missing values processing: Substances of more than 50% missing in comparisons were filtered directly, and substances of less than 50% missing were performed the imputation of missing values using the k-nearest neighbor algorithm. (3) Normalization: The IS or total ion current of each sample was used for the normalization. A total of 1,426 and 293 peaks of the original data were retained for positive and negative ion modes, respectively. The excel sheets, including the name of peak and sample, and the standard data of normalized peak area were obtained for further data analysis.

The peak area of each metabolite was normalized to the area of the internal standard (IS), and subsequent analyses were conducted based on these normalized values. Identified metabolites were annotated using the KEGG COMPOUND database “https://www.kegg.jp/kegg/compound (accessed on 10 February 2022)”, PubChem database, and Human Metabolome Database (HMDB) V4.0. SIMCA (V14.1) software was used for the principal component analysis (PCA) and orthogonal partial-least-squares discriminant analysis (OPLS-DA), and the variable importance projection (VIP) values of metabolites were calculated. All metabolites with VIP values greater than 1.0 and p-values of the Student’s t-test that were less than 0.05 were identified as differential metabolites (DMs) in each paired comparison.

### 2.7 Data preprocessing and annotation

The contents of total sucrose (SC), glucose (GC), fructose (FC), citric acid (CA), and malic acid (MA) in the fruit, as well as the TFC, TPC, TLC, and TFAC in the peel and seed of all samples, were analyzed using ANOVA. All data analyses were performed using SPSS Statistics (Chicago, IL, United States) and expressed as the mean ± standard error of triplicates. Data visualization was conducted using multiple platforms: column charts were generated with GraphPad Prism 9 (v9.5.1, GraphPad Software, San Diego, CA, USA), while Venn diagrams, correlation heatmaps, and butterfly plots were created using OriginPro 2021 (v9.8, OriginLab Corporation, Northampton, MA, USA).

Raw data were converted to the mzXML format using ProteoWizard and processed with an in-house program, which was developed using R and based on XCMS for peak detection, extraction, alignment, and integration. Then, the MS2 database was applied to metabolite annotation. The cutoff for annotation was set at 0.3.

## 3 Results

### 3.1 Comparison of fruit traits of different kumquat varieties

**Figure 1:**
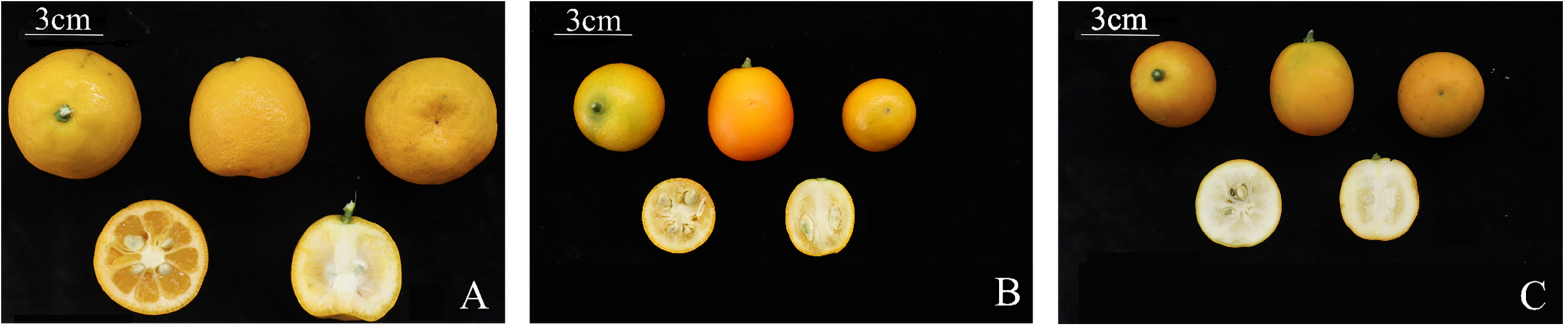
Morphological characteristics of mature kumquat fruit. **(A)** FOT. **(B)** FCSLY. **(C)** FCSHP.

The mature fruits of the three kumquat varieties exhibited significant differences in flavor, oil gland density (**Table 1**), and peel color (**Table 2**). FCSHP was characterized by the sweetest flavor, followed by FCSLY with a slightly sweet taste, while FOT presented an acidic flavor. Both FCSLY and FOT displayed bitterness, whereas FCSHP did not. FOTP exhibited the highest oil gland density, followed by FCSLY and FCSHP (**Table 1**). In terms of fruit color, FCSLY demonstrated the highest gloss brightness value and yellow–blue value, indicating the most pronounced glossiness and deepest yellow hue, respectively. FOT had the highest red–green value, reflecting the most intense red coloration, while FCSHP exhibited the lowest gloss brightness, red–green value, and yellow–blue value (**Figure 1**).

**Table 1:** Kumquat fruit flavor and oil gland density.

Variety name	Variety name code	Mature fruit		
		Fruit flavor	Bitterness	Oil gland density
‘Changshou’ kumquat	FOT	1	1	7
‘Liuyang’ kumquat	FCSLY	7	1	5
‘Huapi’ kumquat	FCSHP	9	0	3

Flesh flavor (1: acid; 7:slightly sweet; 9: sweet); bitterness (0: no; 1: yes); oil gland (3: sparse; 5: medium; 7: dense).

**Table 2:** Kumquat fruit color.

Variety name	Variety name code	L*	a*	b*	CCI
‘Changshou’ kumquat	FOT	66.81 ± 0.52	19.74 ± 0.70	69.05 ± 0.84	4.28 ± 0.15
‘Liuyang’ kumquat	FCSLY	68.11 ± 1.90	16.15 ± 1.75	71.19 ± 2.87	3.35 ± 0.53
‘Huapi’ kumquat	FCSHP	67.96 ± 0.51	15.57 ± 0.90	70.84 ± 1.13	3.23 ± 0.13

The L* value represents gloss brightness, with higher values indicating greater brightness. The a* value represents the red–green value, where larger positive values indicate deeper red tones and smaller negative values indicate deeper green tones. The b* value represents the yellow–blue value, where larger positive values indicate deeper yellow tones and smaller negative values indicate deeper blue tones.

### 3.2 Comparison of metabolite contents in fruits of different kumquat cultivars

#### 3.2.1 Comparison of sugar and acid content in fruit

**Figure 2:**
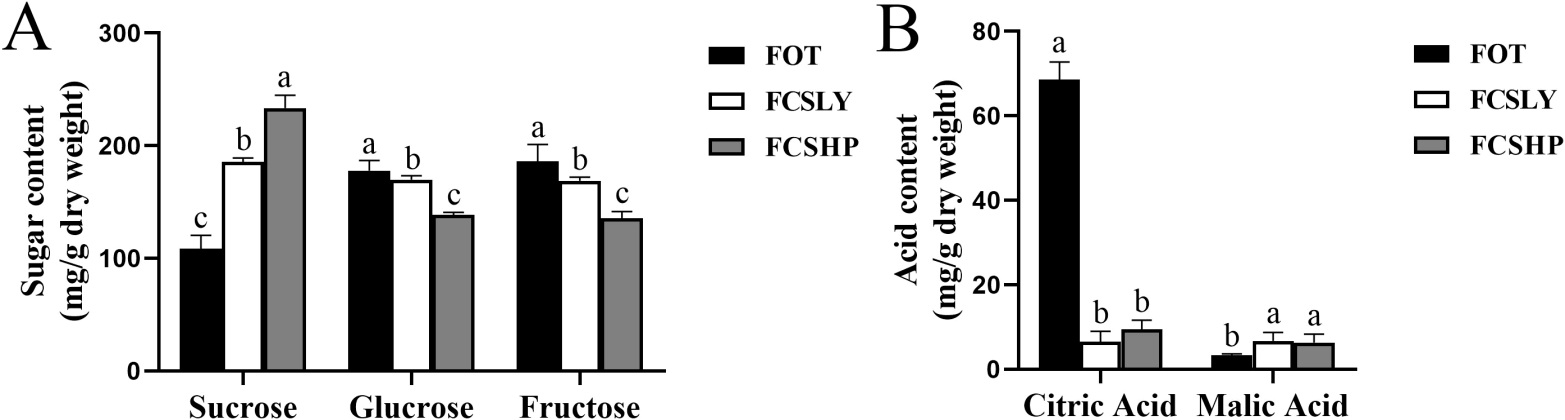
Sugar and acid content of mature kumquat fruit. **(A)** Sugar content in mature kumquat fruits. **(B)** Acid content in mature kumquat fruits. The line represents the average (n = 3), and the error line represents the standard error of the average (SD). Multiple comparisons using the Duncan method revealed significant differences (p < 0.05). No significant difference was observed for labels with the same letters, while histograms with different letters showed significant variations (p < 0.05).

The determination of SC, GC, FC, CA, and MA contents in three mature kumquat varieties revealed significant variations among the groups (**Figure 2**). Regarding SC, the three varieties exhibited distinct differences. FCSHP had the highest SC content, measuring 233.37 ± 11.40 mg/g, followed by FCSLY with 185.57 ± 3.60 mg/g. On the other hand, FOT displayed the lowest SC concentration of 108.46 ± 11.71 mg/g. In terms of GC and FC content, FOT and FCSLY did not differ significantly from each other. Both had higher values compared with FCSHP. Specifically, FOT exhibited the highest GC content of 177.82 ± 9.03 mg/g, followed by FCSLY with 169.77 ± 3.67 mg/g and FCSHP with 138.52 ± 2.41mg/g. Similarly, FOT had the highest FC content of 186.09 ± 14.92 mg/g, followed by FCSLY with 168.77 ± 3.43 mg/g. Furthermore, the CA and MA contents of the three kumquat varieties also displayed significant variations. FOT had the highest CA content of 68.61 ± 4.14 mg/g, followed by FCSHP with 9.42 ± 2.19 mg/g, and FCSLY with 6.49 ± 2.46 mg/g. In terms of MA content, FCSLY had the highest value of 6.73 ± 1.97 mg/g, followed by FCSHP with 6.32 ± 1.99 mg/g, and FOT with 2.31 ± 0.78 mg/g.

#### 3.2.2 Comparison of secondary metabolite content in fruit peel and seeds

**Figure 3:**
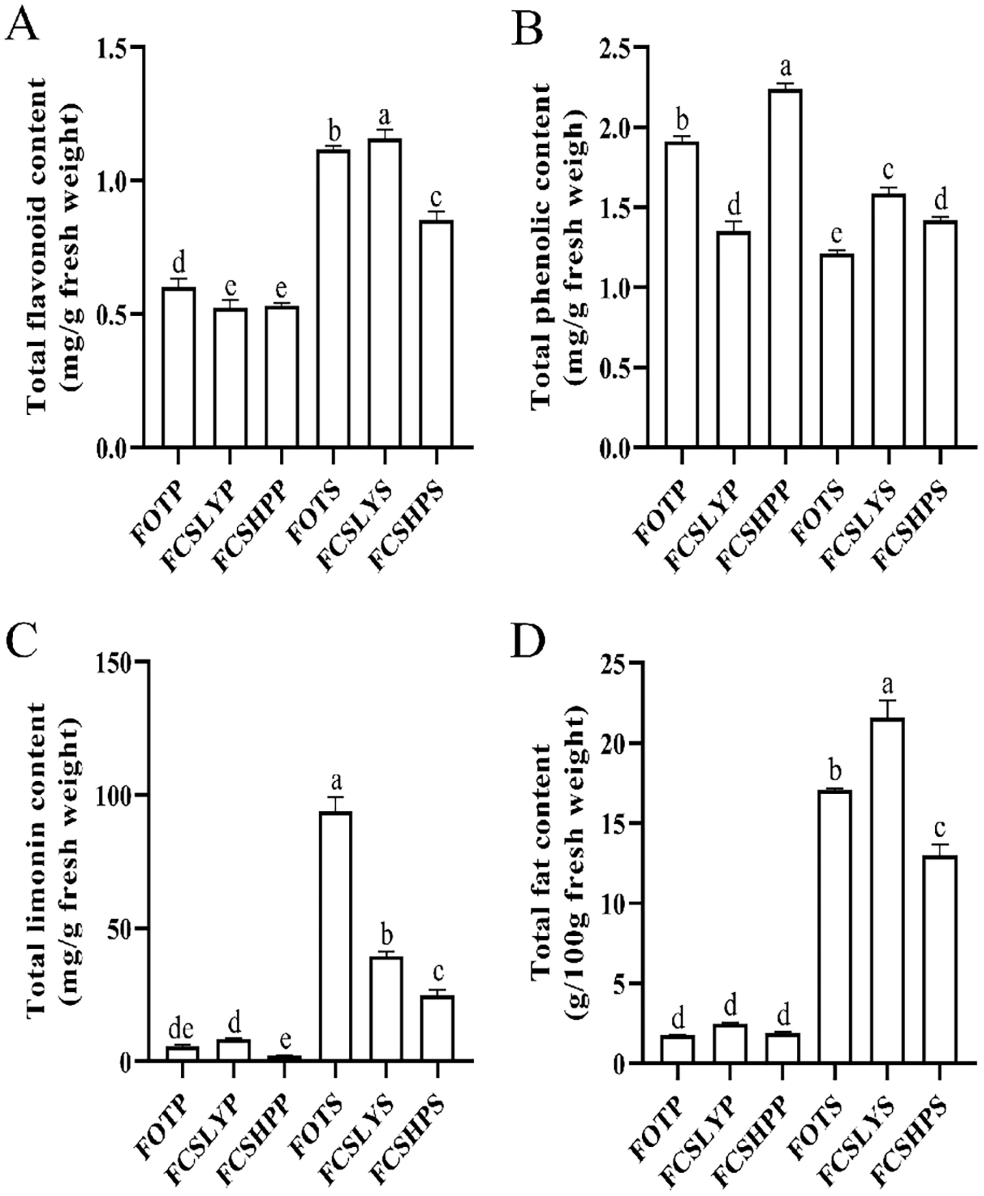
Secondary Metabolite Content in Three Kumquat Varieties. The figure illustrates the content of secondary metabolites in three kumquat varieties, namely FOTP, FCSLYP, and FCSHPP. The metabolites analyzed include TFC **(A)**, TPC **(B)**, TLC **(C)**, and TFAC **(D)**. Each data point represents the average value from three replicates, and the error bars indicate the SD. To assess the significance of differences, multiple comparisons were performed using the Duncan method, with a significance level of *p* < 0.05. Labels with the same letters indicate no significant difference, while different letters on the histograms indicate significant differences (*p* < 0.05).

The peels and seeds of three kumquat varieties were analyzed to determine the TFC, TPC, TLC, and TFAC (**Figure 3**). These examined parameters showed significant variations among the peels of the three kumquat varieties. FOTP exhibited a higher TFC (0.60 ± 0.03 mg/g) compared with FCSLYP (0.52 ± 0.03 mg/g) and FCSHPP (0.53 ± 0.01 mg/g). FCSHPP had the highest TPC (2.24 ± 0.03 mg/g), followed by FOTP (1.91 ± 0.03 mg/g) and FCSLYP (1.35 ± 0.06 mg/g). FCSLYP had the highest TLC (8.43 ± 0.38 mg/g), followed by FOTP (5.66 ± 0.63 mg/g) and FCSHPP (2.24 ± 0.04 mg/g). There was no significant difference in TFAC among the three varieties, ranging from 1.77 ± 0.04 to 2.48 ± 0.08 g/100 g. In the comparison of seed groups, all four secondary metabolite content indices showed significant differences. FCSLYS had significantly higher TFC (1.16 ± 0.03 mg/g), TPC (1.58 ± 0.04 mg/g), and TFAC (21.58 ± 1.09 g/100 g) compared with FOTS and FCSHPS. FOTS (93.99 ± 5.38 mg/g) exhibited significantly higher TLC compared with the other two comparison groups. When comparing kumquat peels with seeds, the TFC, TLC, and TFAC were all significantly higher in kumquat seeds, while the TPC was higher in kumquat peels. To gain further insights into the major metabolite compositions of these varieties, we investigated the metabolomic profiles of the three kumquat peels and seeds.

### 3.3 Comparative metabolomic analysis of kumquat varieties: peel and seed comparison

#### 3.3.1 Multivariate statistical analysis

**Figure 4:**
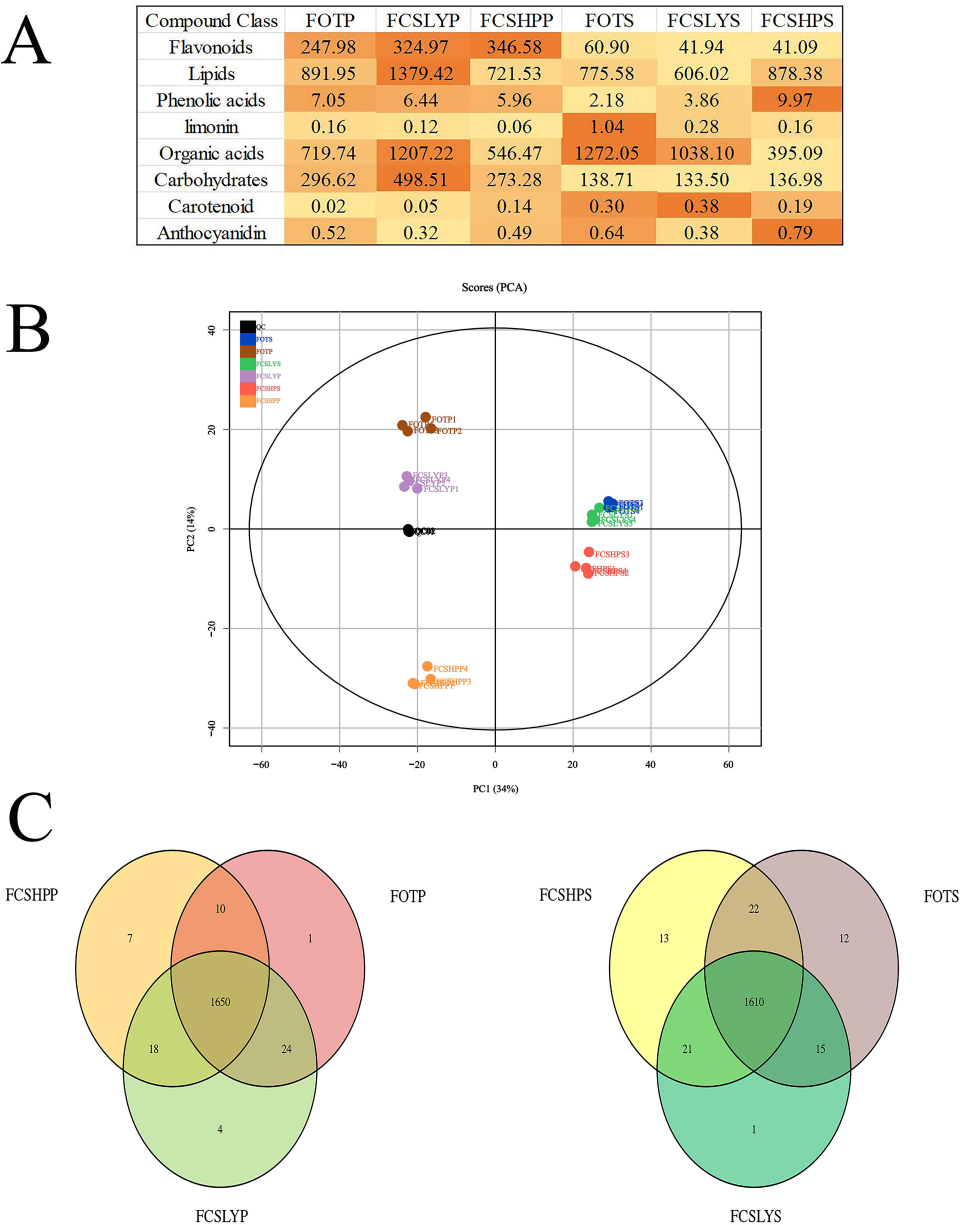
Overview of metabolic analysis detected in the peels and seeds of three kumquats. **(A)** The strength of each compound type. The color intensity in Figure **(A)** represents the relative content of each compound. **(B)** Principal component analysis of peel and seed metabolites of three kumquat varieties. **(C)** The Venn diagram of the distribution of seed metabolites in three kumquat varieties.

The identification of primary and secondary metabolites in the samples was carried out using the UPLC-MS platform, employing non-targeted metabolomics technology. A comprehensive analysis led to the detection of a total of 1719 metabolites (**Supplementary Tables S1**, **S2**). These metabolites can be categorized into 15 compound classes, encompassing 161 flavonoids, 32 coumarins, 28 cinnamic acids, 25 lignans, 32 nucleotides, 175 organic acids, 511 lipids, 115 amino acids and their derivatives, 114 terpenes, 4 tannins, 38 alkaloids, 49 phenols, 44 phenolic acids, 151 carbohydrates, and 253 other compounds. The sum of all compounds within each class differed among the kumquat variety and its corresponding parts (**Figure 4A**), such that FCSHPP was rich in flavonoids, FCSLYP in lipids and carbohydrates, FOTS in limonin and organic acids, FCSLYS in carotenoids, and FCSHPS in anthocyanins and phenolic acids. These results indicate that each sample has unique nutritional and medicinal values.

The principal component analysis (PCA) results indicated that the four replicates of each sample clustered closely together (**Figure 4B**). Additionally, there was a clear separation between different samples, suggesting strong data repeatability and stability. Moreover, the metabolites in different parts of the various kumquat varieties exhibited significant differences, consistent with their observed phenotypic variations. The first principal component (PC1) accounted for 34% of the variability in the original dataset. Analysis of the PC1 revealed a distinct separation of peel and seed data points, while variations between different varieties were observed on the second principal component, explaining 14% of the dataset’s variance. All samples were classified into two categories, with FCSHPS, FCSLYS, and FOTS clustering together, similar to FCSHPP, FCSLYP, and FOTP. This clustering pattern aligned with the PCA plot results, suggesting that disparities in metabolites may contribute to the observed differences in fruit phenotypes among the kumquat varieties. The Venn diagram (**Figure 4C**) illustrated that the peel and seed comparison groups of the three varieties contained 1650 and 1610 metabolites, respectively, with relatively few differing metabolites between the groups. This indicates a high degree of similarity in metabolite composition across the three kumquat varieties.

#### 3.3.2 Analysis of differential metabolites

**Figure 5:**
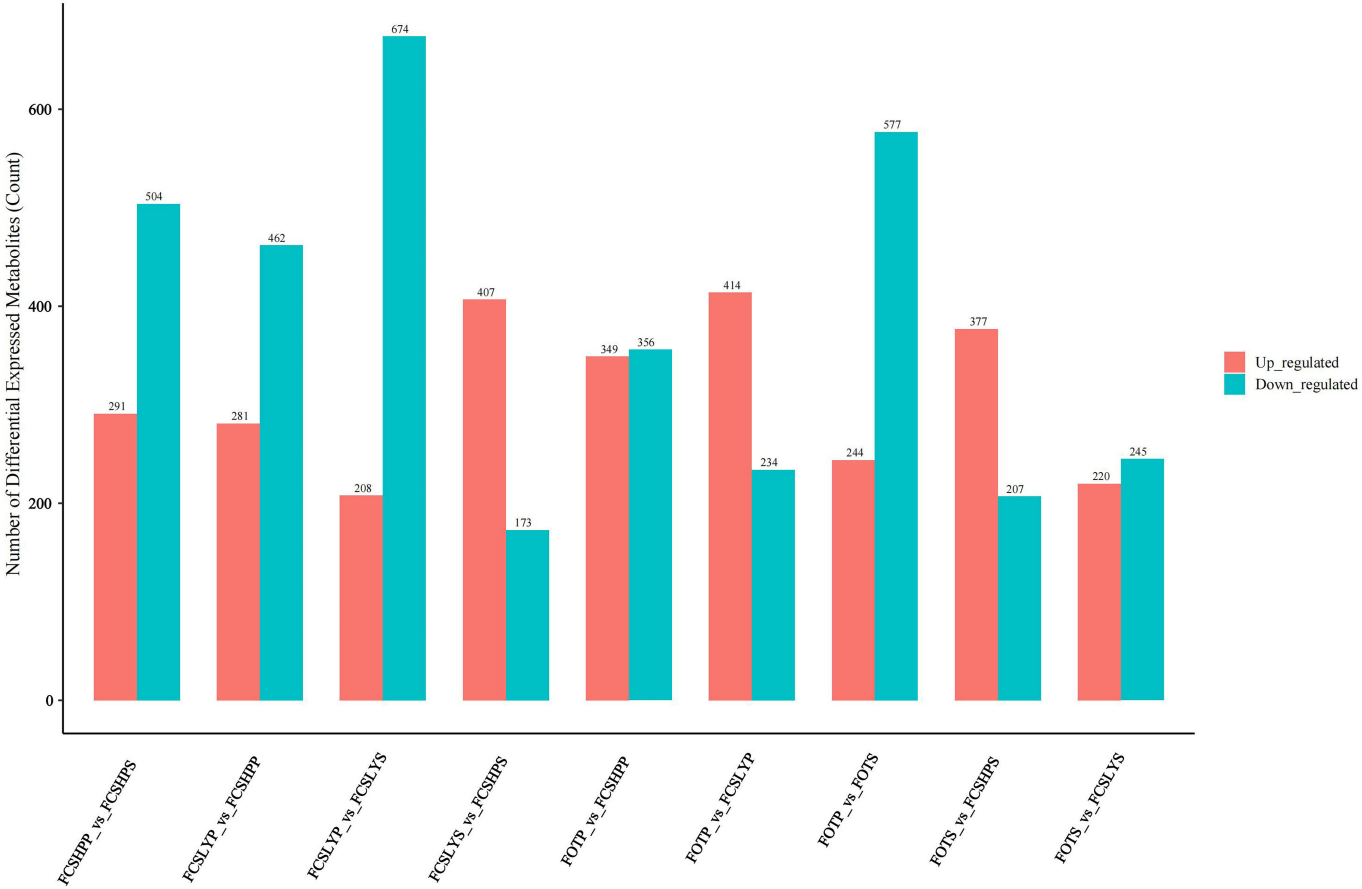
Statistical histogram of differential metabolites.

We conducted a differential metabolite analysis of pairwise comparisons among the peel and seed of three different varieties of kumquats, as well as the peel and seed within the same variety, resulting in a total of nine comparison groups. The differential metabolites were screened using Student’s t-test *p*-values and the variable importance in projection (VIP) values from the OPLS-DA model. Metabolites with a *p*-value less than 0.01 were considered statistically significant, and the results of this screening process are presented as a histogram (**Figure 5**).

In the comparison between the FCSHPP and FCSHPS groups, a total of 791 differential metabolites were identified, among which 291 were up-regulated and 504 were down-regulated. The comparative analyses for other groups are presented in **Figure 5**. Specifically, in the peel comparison group, FCSLYP exhibited the highest number of up-regulated metabolites, with 159 identified as such. Conversely, in the seed comparison group, FCSHPS displayed the highest count of up-regulated metabolites. In the comparison of peels and seeds, all three varieties demonstrated a greater number of up-regulated metabolites in the peel. These findings indicate that FCSLYP and FCSHPS possess a higher metabolite abundance and greater medicinal value, with the peel exhibiting a higher medicinal value than the seeds.

#### 3.3.3 Differential accumulation of flavonoids and phenolic acids metabolites

**Figure 6:**
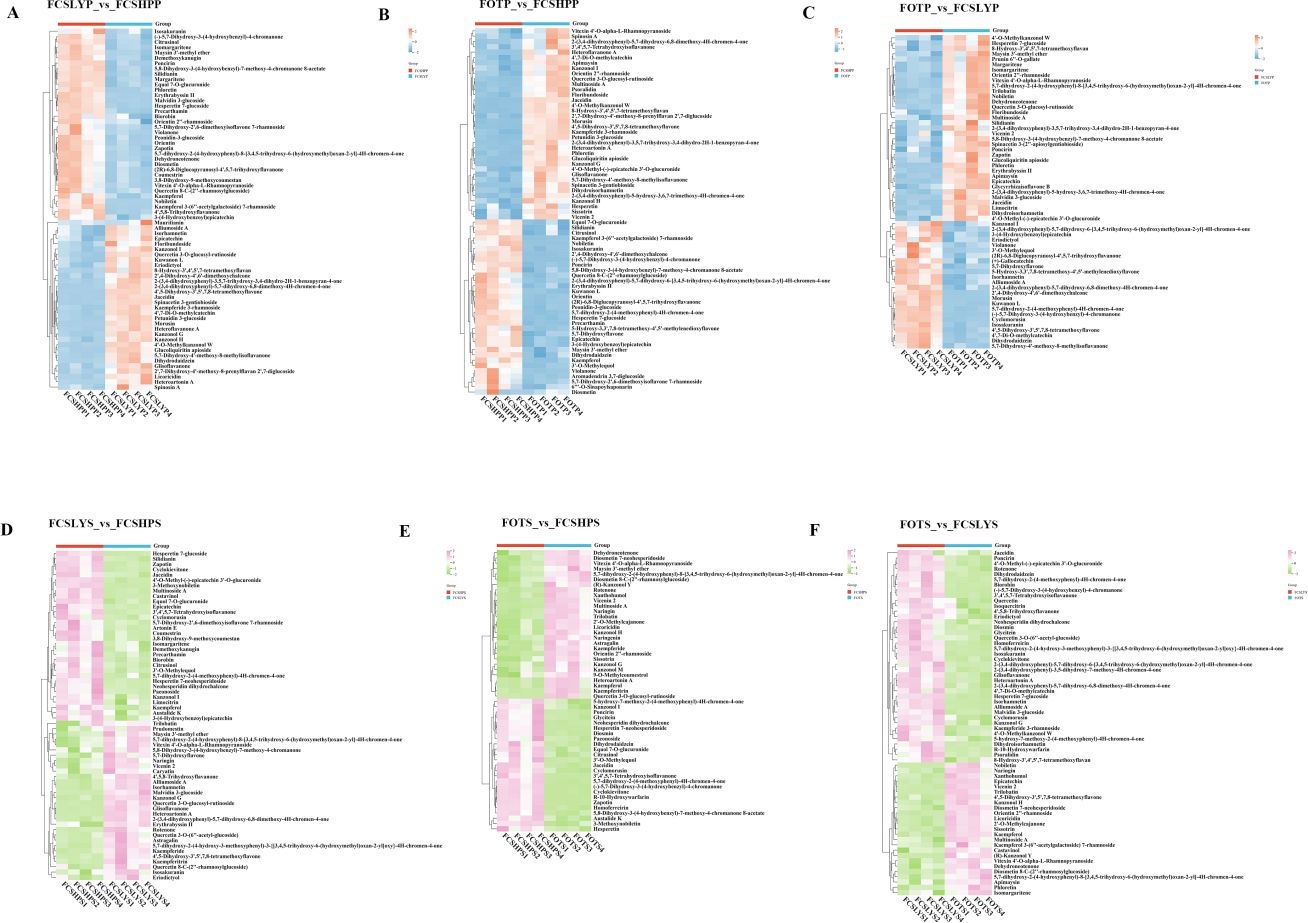
Heat map visualization of the relative content of differential flavonoid metabolites in the peel and seed comparison groups: **(A)** FCSLYP_vs_FCSHPP; **(B)** FOTP_vs_FCSHPP; **(C)** FOTP_vs_FCSLYP; **(D)** FCSLYS_vs_FCSHPS; **(E)** FOTS_vs_FCSHPS; **(F)** FOTS_vs_FCSLYS; Each column represents one sample. Each column represents one sample, and each row represents one metabolite. In the peel comparison groups, the color change from orange to blue indicates abundance changes from high to low; in the seed comparison groups, the color change from pink to green indicates abundance changes from high to low.

This study conducted a comprehensive analysis of flavonoid and phenolic acid metabolites in the peels and seeds of three kumquat varieties. For flavonoid metabolites, most compounds were found in glycosylated forms, primarily classified as O-glycosides and C-glycosides. The major glycosylation sites were at 3-O, 5-O, 7-O (O-glycosides) and 6-C, 8-C (C-glycosides), while O-glycosides at 6-OH or 8-OH positions were less common. The glycosyl groups included glucose, rhamnose, rutinose, and neohesperidose. In peel comparisons, FOTP showed higher accumulation of diverse flavonoids (**Figures 6B, C**). Examples include O-glycosylated flavonoids such as Vitexin 4’-O-α-L-rhamnopyranoside and Quercetin 3-O-glucosyl-rutinoside, flavanones like 2-(3,4-dihydroxyphenyl)-5-hydroxy-3,6,7-trimethoxy-4H-chromen-4-one, and flavanols such as Epicatechin. FCSLYP showed increased accumulation of both methylated flavonoids (e.g., 4’,5-dihydroxy-3’,5’,7,8-tetramethoxyflavone) and upregulated isoflavones (e.g., 5,7-dihydroxy-4’-methoxy-8-methylisoflavanone and dihydrodaidzein) (**Figures 6A, C**). FCSHPP exhibited significant accumulation of polymethoxyflavones (PMFs) including Nobiletin, Diosmetin, Poncirin, and Hesperetin 7-glucoside, as well as upregulated C-glycosylated flavonoids and their derivatives like Orientin and Quercetin 8-C-(2’’-rhamnosylglucoside) (**Figures 6A, B**). In seed comparisons, FOTS primarily upregulated C-glycosylated flavonoids (Vicenin 2, Vitexin 4’-O-α-L-Rhamnopyranoside, Orientin 2’’-rhamnoside) and O-glycosylated flavonoids (Naringin, Diosmetin 7-neohesperidoside, Diosmetin 8-C-(2’’-rhamnosylglucoside)), along with the prenylated flavonoid Xanthohumol (**Figures 6E, F**). FCSLYS was characterized by methylated flavonoids (Isorhamnetin) and flavanol derivatives (4’-O-Methyl-epicatechin 3’-O-glucuronide), and contained isoflavones such as 3’,4’,5,7-Tetrahydroxyisoflavanone (**Figures 6D, F**). FCSHPS displayed a unique accumulation pattern of PMFs, including 3-Methoxynobiletin and Zapotin (**Figures 6D, E**).

**Figure 7:**
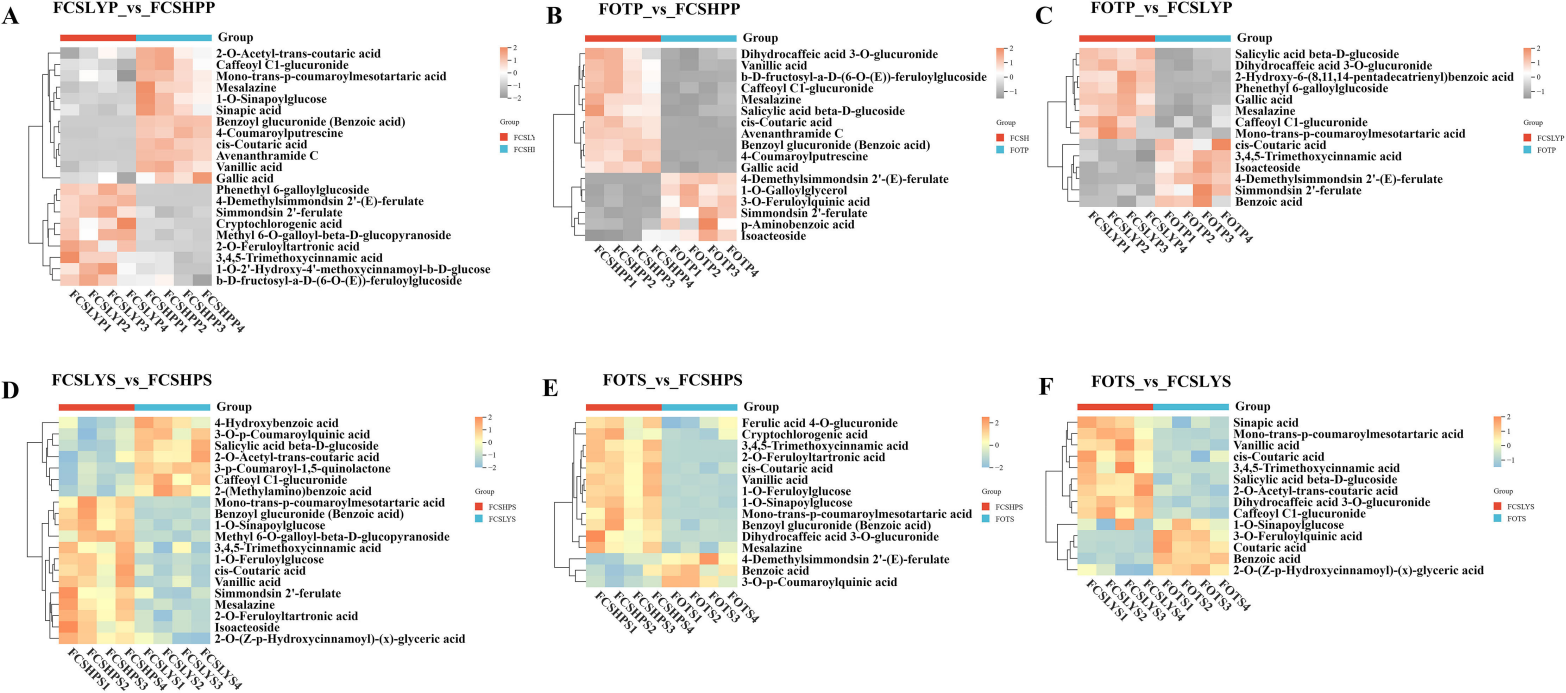
Differential phenolic acid metabolites in the peel and seed comparison groups **(A)** FCSLYP_*vs*_FCSHPP; **(B)** FOTP_*vs*_FCSHPP; **(C)** FOTP_*vs*_FCSLYP; **(D)** FCSLYS_*vs*_FCSHPS; **(E)** FOTS_*vs*_FCSHPS; **(F)** FOTS_*vs*_FCSLYS; Each column represents one sample, and each single row represents one metabolite. In the peel comparison groups, orange to gray indicates abundance changes from high to low; in the seed comparison groups, orange-yellow to blue indicates abundance changes from high to low.

Higher levels of phenolic acids were accumulated in both FCSHPP and FCSHPS (**Figure 7**). FOTP predominantly accumulated 4-Demethylsimmondsin 2’-(E)-ferulate, Simmondsin 2’-ferulate, and Isoacteoside (**Figures 7B, C**). In FCSLYP, Phenethyl 6-galloylglucoside showed significant upregulation (**Figures 7A, C**). FCSHPP accumulated various phenolic acid metabolites including Gallic acid, 4-Coumaroylputrescine, Caffeoyl glucuronide, Avenanthramide C, and Mesalazine (**Figures 7A, B**). In seed samples, Benzoic acid was highly accumulated in FOTS (**Figures 7E, F**). The upregulated metabolites in FCSLYS included Caffeoyl C1-glucuronide, 2-O-Acetyl-trans-coutaric acid, and Salicylic acid beta-D-glucoside (**Figures 7D, F**). FCSHPS exhibited the most complex phenolic acid profile (**Figures 7D, E**), with multiple compounds detected including Vanillic acid, Mesalazine, Mono-trans-p-coumaroylmesotartaric acid, 1-O-Feruloylglucose, 2-O-Feruloyltartronic acid, 1-O-Sinapoylglucose, cis-Coutaric acid, 3,4,5-Trimethoxycinnamic acid, and Benzoyl glucuronide.

### 3.4 Association analysis between secondary metabolites and fruit traits in kumquat fruit

**Figure 8:**
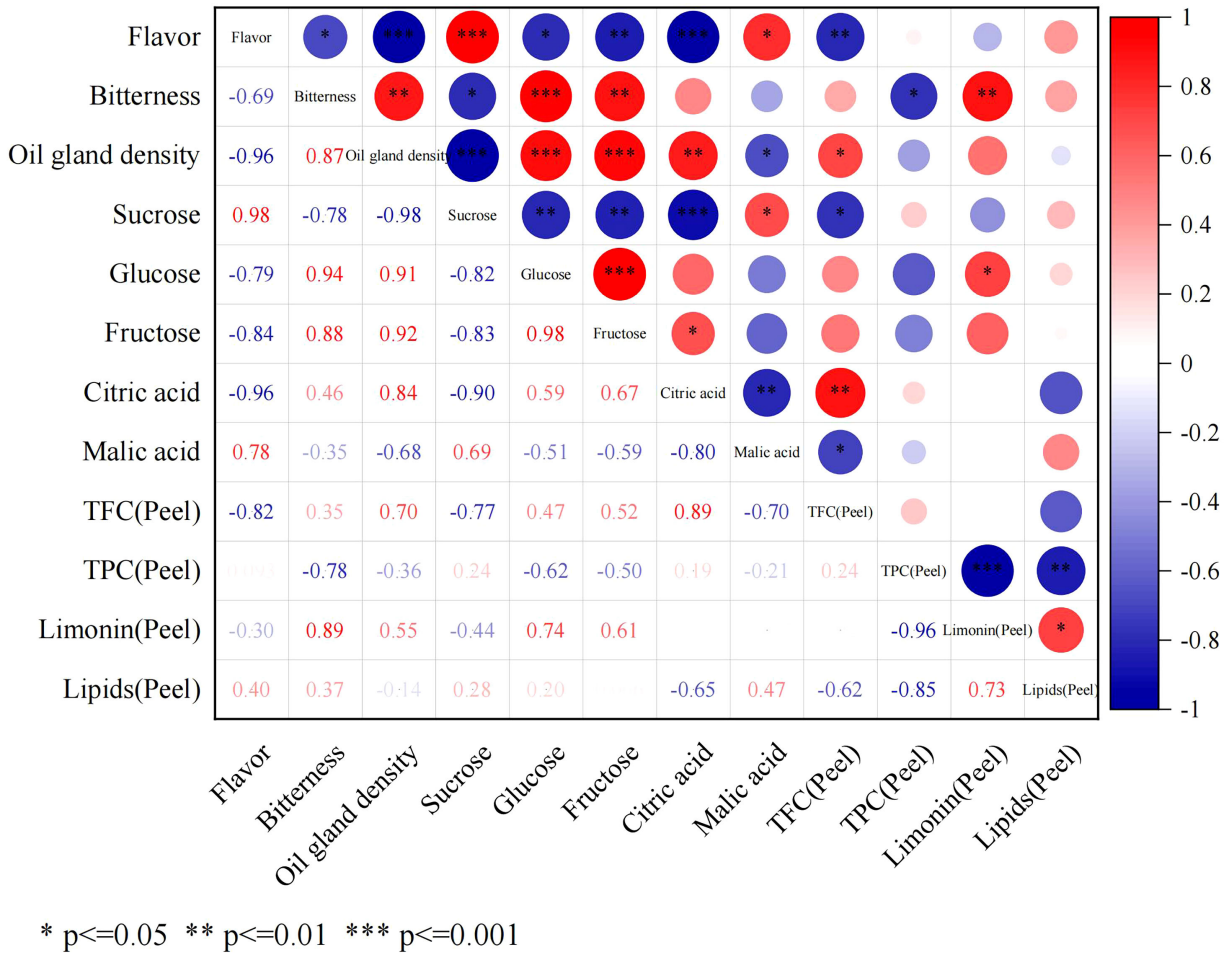
Correlation matrix between fruit flavor and all chemical indexes.

The correlation between phenotypic traits (fruit flavor, bitterness, and oil gland density) and fruit sugar and acid content (SC, GC, FC, CA, and MA contents) and peel secondary metabolite contents (total flavonoids, total phenols, total limonin, and total lipids) was investigated (**Figure 8**). The findings revealed that fruit flavor exhibited a positive correlation with SC content (r = 0.98) and a negative correlation with oil gland density and CA content (r = −0.96). Fruit bitterness demonstrated a strong positive correlation with oil gland density (r = 0.87) and TLC (r = 0.89) in the peel.

#### 3.4.1 Correlation analysis between secondary metabolites and fruit flavor

FCSHP exhibited a sweet flavor without bitterness. FCSLY had a sweet and bitter flavor, while FOT had a sour and bitter taste (**Table 1**). We conducted measurements on the sugar and acid content of the entire fruit, and the SC content in the sweet FCSHP was significantly higher than in the other two varieties, reaching 233.37 ± 11.40 mg/g. The next highest SC content was found in the sweet FCSLY, whereas FOT had the lowest SC concentration (**Figure 2A**). A significant correlation was identified between fruit flavor and SC content (r = 0.98) (**Figure 8**), indicating that SC content is a crucial chemical indicator for determining the sweetness of kumquat. Regarding the flavor of FOT, it is characterized as acidic and it has the highest MA content. The MA content makes the fruit have sour taste characteristics. In terms of bitterness, FCSLYP exhibited the highest limonin content, followed by FOTP, with FCSHPP displaying the lowest content (**Figure 3C**). This result is consistent with the relative content of limonin in the three varieties and corroborates the phenotypic characteristic that FOT and FCSLY fruits exhibit bitterness, whereas FCSHP does not. Furthermore, a negative correlation was observed between fruit bitterness and SC content (correlation coefficient = −0.72) (**Figure 8**). The limonin content in the peel emerged as a crucial chemical indicator affecting fruit bitterness. Consequently, FCSHP is deemed more suitable for consumption as whole fruit fresh food.

#### 3.4.2 Correlation analysis of secondary metabolites and pericarp oil gland

**Figure 9:**
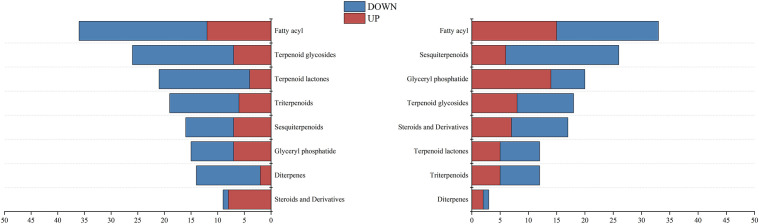
Differential expression of lipid metabolites between kumquat peel comparison groups.

The oil gland density in the peel of the three kumquat varieties followed the order: FCSHPP < FCSLYP < FOTP. However, there was no significant difference in TFAC among the three varieties (**Figure 3D**). Analysis of non-targeted metabolomics data revealed that FCSHPP exhibited a greater number of down-regulated lipid compounds compared with FOTP and FCSLYP (**Figure 9**). Additionally, the relative content of lipid compounds was also lower in FCSHPP, which corresponded to the observed oil gland density (**Figure 4A**). Terpenes and sesquiterpenes are the main constituents of citrus essential oils found in lipids. It is plausible that these down-regulated lipids are key metabolites highly correlated with oil gland density (**Table 3**).

**Table 3:** Terpene and sesquiterpene metabolites down-regulated by FCSHPP.

Compound	Class	Compound	Class
10-Hydroxy-3-methoxy-1,3,5,7-cadinatetraen-9-one	Sesquiterpenoids	Isohumbertiol	Sesquiterpenoids
Diacetoxyscirpenol	Sesquiterpenoids	(1alpha,6alpha,7alphaH)-2,4-(15)-Copadiene	Sesquiterpenoids
Armillarin	Sesquiterpenoids	8beta-Angeloyloxy-15-hydroxy-1alpha,10R-dimethoxy-3-oxo-11-(13)-germacren-12,6alpha-olide	Terpene lactones
Lacinilene C	Sesquiterpenoids	Anhydrocinnzeylanol	Terpene lactones
3,7-Bisaboladiene-2,8-dione	Sesquiterpenoids	Eremopetasitenin C3	Terpene lactones
8-Oxodiacetoxyscirpenol	Sesquiterpenoids	Cadabicilone	Terpene lactones
Dehydrovomifoliol	Sesquiterpenoids	Veranisatin C	Terpene lactones
beta-Vatirenene	Sesquiterpenoids	(-)-trans-Carveol glucoside	Terpene glycosides
(1R,3R,4R,5S,6S,8x)-1-Acetoxy-8-angeloyloxy-3,4-epoxy-5-hydroxy-7 (14),10-bisaboladien-2-one	Sesquiterpenoids	Nepetaside	Terpene glycosides
Santalyl phenylacetate	Sesquiterpenoids	Perilloside A	Terpene glycosides
(+)-4,11-Eudesmadien-3-one	Sesquiterpenoids	Deoxyloganic acid	Terpene glycosides
Epioxylubimin	Sesquiterpenoids	(4R,5S,7R,11S)-11,12-Dihydroxy-1(10)-spirovetiven-2-one 11-glucoside	Terpene glycosides
Furoeremophilone 1	Sesquiterpenoids	(1S,4R)-10-Hydroxyfenchone glucoside	Terpene glycosides

#### 3.4.3 Secondary metabolites and peel color

The non-targeted metabolomics results revealed that FCSHPP, with a lighter fruit peel color, exhibited up-regulated expression of the 3-(4-Hydroxybenzoyl) epicatechin metabolite only compared with FCSLYP (**Table 4**). Additionally, FCSHPP demonstrated the highest carotenoid abundance (**Figure 4A**). Conversely, FOTP, characterized by the strongest redness, demonstrated a greater up-regulated expression of anthocyanin and carotene metabolites in comparison with the other two groups (**Table 4**), with the highest anthocyanin content (**Figure 4A**). Furthermore, compared with FCSHPP, FCSLYP exhibited a higher up-regulation of anthocyanin metabolites (**Table 4**). Consequently, it can be inferred that anthocyanin and carotene metabolites are associated with the color of kumquat peel. Notably, the metabolomic results aligned with the fruit color phenotype, with FOTP displaying the strongest redness, FCSLYP showcasing the brightest color brightness and strongest yellowness, and FCSHPP displaying the lowest brightness, redness, and yellowness (**Table 2**).

**Table 4:** Comparison of anthocyanin and carotenoid metabolites in three peel comparison groups.

Compound	FCSLYP*vs*FCSHPP	FOTP*vs*FCSHPP	FOTP*vs*FCSLYP
anthocyanin			
(+)-Gallocatechin	ns	ns	0.026
3-(4-Hydroxybenzoyl)epicatechin	1.251	0.003	0.014
4’,7-Di-O-methylcatechin	0.003	0.002	0.014
4’-O-Methyl-(-)-epicatechin 3’-O-glucuronide	NS	0.009	0.023
Epicatechin	0.026	0.008	0.012
Malvidin 3-glucoside	0.003	NS	0.036
Petunidin 3-glucoside	0.007	0.001	0.036
carotenoid			
Lutein	ns	0.081	ns
(9Z,7’Z,9’Z)-7,8-Dihydrolycopene	ns	ns	ns
Phytofluene	ns	ns	ns

The values presented in the table represent the FOLD CHANGE (FC) values. The fold change is calculated based on the ratio of the peak areas of each metabolite in the comparative group samples. For instance, in the comparison a versus b, the FC is determined by the formula Fc = b/a, where ‘a’ and ‘b’ correspond to the average peak areas of the metabolite from four replicates of the left and right samples in the comparative group, respectively. The value of FC>1 indicates that the expression of metabolites on the right side of the control group is up-regulated; The abbreviation “ns” denotes that the metabolite in question is not identified as a differential metabolite within the control group.

### 3.5 Differences of functional components of flavonoids and limonin between peels and seeds

In addition to the TPC, the TFC, TLC, and TFAC were found to be significantly higher in the seeds of the three kumquat varieties compared with the peels (**Figure 3**). Conversely, the peel exhibited a higher abundance of flavonoid metabolites. Analysis of these three varieties revealed that 35 flavonoid metabolites were up-regulated in the peel, while 18 flavonoid metabolites were up-regulated in the seeds (**Table 5**). Hence, it can be inferred that the peel possesses a greater medicinal value.

**Table 5:** Comparison of up-regulated flavonoid metabolites in the peels and seeds of three kumquat cultivars.

Compound	Class	Compound	Class
peel			
Phloretin	Chalcones and dihydrochalcones	Diosmetin 8-C-(2’’-rhamnosylglucoside)	Flavonoid glycosides
epsilon-Viniferin	2-arylbenzofuran flavonoids	Margaritene	Flavonoid glycosides
(-)-5,7-Dihydroxy-3-(4-hydroxybenzyl)-4-chromanone	Homoisoflavonoids	Isoquercitrin	Flavonoid glycosides
5,8-Dihydroxy-3-(4-hydroxybenzyl)-7-methoxy-4-chromanone 8-acetate	Homoisoflavonoids	Isomargaritene	Flavonoid glycosides
5,7-dihydroxy-2-(4-methoxyphenyl)-4H-chromen-4-one	Flavonoids	Spinacetin 3-gentiobioside	Flavonoid glycosides
Cyclomorusin	Flavonoids	(2R)-6,8-Diglucopyranosyl-4’,5,7-trihydroxyflavanone	Flavonoid glycosides
Dihydroisorhamnetin	Flavonoids	Quercetin	Flavonoids
8-Hydroxy-3’,4’,5’,7-tetramethoxyflavan	Flavonoids	Isorhamnetin	Flavonoids
Heteroflavanone A	Flavonoids	5,7-Dihydroxyflavone	Flavonoids
Citrusinol	Flavonoids	Silidianin	Flavans
Vitexin 4’-O-alpha-L-Rhamnopyranoside	Flavonoid glycosides	Kanzonol I	Isoflavonoids
Kaempferide 3-rhamnoside	Flavonoid glycosides	4’-O-Methylkanzonol W	Isoflavonoids
Hesperetin 7-glucoside	Flavonoid glycosides	Dehydroneotenone	Isoflavonoids
Isosakuranin	Flavonoid glycosides	Violanone	Isoflavonoids
5,7-dihydroxy-2-(4-hydroxyphenyl)-8-[3,4,5-trihydroxy-6-(hydroxymethyl)oxan-2-yl]-4H-chromen-4-one	Flavonoid glycosides	Equol 7-O-glucuronide	Isoflavonoids
Alliumoside A	Flavonoid glycosides	Dihydrodaidzein	Isoflavans
Apimaysin	Flavonoid glycosides	3,8-Dihydroxy-9-methoxycoumestan	Isoflavonoids
2-(3,4-dihydroxyphenyl)-5,7-dihydroxy-6-[3,4,5-trihydroxy-6-(hydroxymethyl)oxan-2-yl]-4H-chromen-4-one	Flavonoid glycosides		
Seed			
R-10-Hydroxywarfarin	Homoisoflavonoids	Castavinol	Flavans
4’,5-Dihydroxy-3’,5’,7,8-tetramethoxyflavone	Flavonoids	Epicatechin	Flavans
Zapotin	Flavonoids	Licoricidin	Isoflavonoids
Poncirin	Flavonoid glycosides	Kanzonol H	Isoflavonoids
Maysin 3’-methyl ether	Flavonoid glycosides	3’-O-Methylequol	Isoflavonoids
Biorobin	Flavonoid glycosides	2’-O-Methylcajanone	Isoflavans
Heteroartonin A	Flavonoids	Psoralidin	Isoflavonoids
Kaempferol	Flavonoids	(R)-Kanzonol Y	Chalcones and dihydrochalcones
KB 2	Flavonoids	Viniferal	2-arylbenzofuran flavonoids

**Figure 10:**
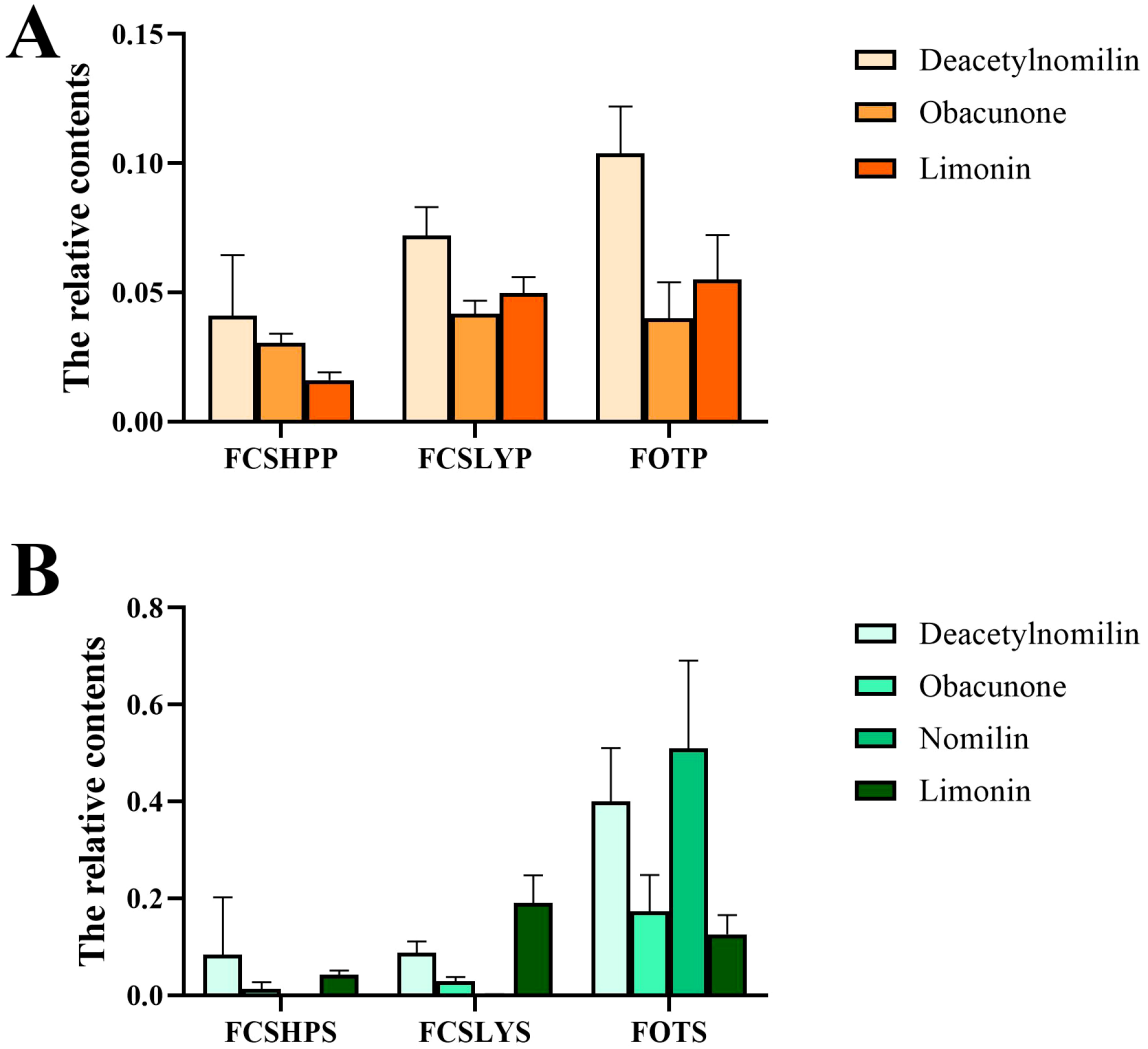
Relative abundance of metabolites in peels and seeds of three kumquats, analyzed using LC-MS/MS and HPLC. **(A)** Relative content of limonoid metabolites in the peel. **(B)** Relative content of limonoid metabolites in the seeds.

The kumquat seeds were found to contain higher levels of limonin compounds compared with peels, particularly with the TLC reaching 93.99 ± 5.38 mg/g in FOTS. Non-targeted metabolomics analysis detected four limonin compounds, namely deacetylnomilin, limonin, Obacunone, and nomilin. In the peel samples, Deacetylnomilin, Obacunone, and Limonin were detected in all three varieties (FCSHPP, FCSLYP, and FOTP) (**Figure 10A**), with FOTP showing the highest accumulation and FCSHPP the lowest. In seeds, in addition to these three limonoids, Nomilin was also detected (**Figure 10B**). All four limonoid metabolites accumulated most abundantly in FOTS. This suggests that kumquat seeds can be utilized as a raw material for extracting limonin.

## 4 Discussions

Various factors influence the flavor of kumquat fruit, and the SC content plays a crucial role in determining its sweetness. Limonoids contribute to the bitter taste in kumquats. Additionally, flavonoid metabolites, such as poncirin, naringin, and nobiletin, also contribute to the bitterness of citrus fruits. In our study, the aforementioned three metabolites were found to be up-regulated in FCSHPP. Furthermore, the accumulation of limonoid compounds was lowest in FCSHPP, which is consistent with the findings reported by Qiaoli Ma et al (Ma et al., 2021), indicating that limonoids are the main factors affecting bitterness. Naringin can be hydrolyzed to L-rhamnose and chernoside, with the bitterness reduced through further hydrolysis of 33% of the chernoside of naringin to non-bitter naringenin and D-glucose. However, naringenin is a unique FCSHPP flavonoid metabolite. We also observed the up-regulation of neohesperidin dihydrochalcone, an important sweetening compound, in addition to the highest SC concentration in FCSHP. These differences in metabolite underscore the reason why FCSHP imparts a pleasant taste, making it more suitable for fresh consumption of whole fruit.

The pigmentation of the peel is a crucial aspect of fruit appearance, as consumers make their selection based on factors such as red and yellow intensity, luminosity, and peel pigmentation (Ze et al., 2012). In citrus fruits, the peel color is primarily determined by water-soluble anthocyanins and fat-soluble carotenes (Xu and Deng, 2002). The peel color of the three kumquat varieties exhibited distinct differences. FOTP displayed the highest levels of redness and color index, with a greater abundance of up-regulated anthocyanin and carotene metabolites. FCSLYP exhibited superior color brightness and increased yellowness. In contrast, FCSHPP demonstrated the lowest color index, indicating that the anthocyanin and carotene metabolites impacted the kumquat peel coloration.

Lipids serve as key components or precursors of essential oils, primarily synthesized and stored in the oil glands of citrus fruits (Siau Sie et al., 2012). Sesquiterpenes and terpenes are the primary constituents of essential oils (Flamini et al., 2007). The oil gland density on the surface of FCSHP is the lowest, while the surface of FCSLY and FOT has a higher oil gland density. However, there was no significant difference in the TFAC among the three kumquat varieties. Nevertheless, the metabolomic analysis revealed that FOTP and FCSLYP exhibited a greater abundance of up-regulated differential metabolites in steroids, sesquiterpenes, triterpenoids, terpene lactones, terpenoid glycosides, and fatty acyl subclasses, compared with FCSHPP. These metabolites may be associated with the characteristic of oil gland density. Consequently, the oil gland density in kumquats appears unrelated to the total lipid content in the peel but influenced by specific lipid metabolites.

Limonoids, which are tetracyclic triterpenoids, represent a major source of bitterness in citrus plants. They exhibit a wide range of biological activities, including anticancer, antibacterial, antiviral, antioxidant, and insecticidal properties (Shunming et al., 2019). Bently et al. demonstrated that limonoids derived from citrus plants possess certain anti-feeding effects on Lepidoptera, Coleoptera, and Heteroptera (Bentley et al., 1990). Additionally, other studies have reported their inhibitory effects on insect growth (Essoung et al., 2018; Jimenez et al., 1997). The content of limonoids in FCSLYP and FOTP is higher compared with FCSHPP, which enhances their resistance to insects and diseases. Liu et al. (Can et al., 2012) reported the extraction yield of limonin from citrus seeds to be 7.5 mg/g under optimal conditions. Vaniya Chinapongtitiwat et al. reported limonin contents in the peels of pomelo and lime to be 86 ± 10 ppm and 194 ± 34 ppm (DW), respectively. (Montoya et al., 2019) reported the TLC in the seeds of citrus, lemon, sweet orange, and sour orange, which were 7497 ± 870 mg/kg, 5349 ± 112 mg/kg, 4899 ± 385 mg/kg, and 1035 ± 140 mg/kg, respectively. Ma detected the TLC in Nanfeng mandarin peel within the range of 3.08 ± 0.07 to 5.51 ± 0.11 mg/g DW (Ma, 2019). The findings of this study revealed that the TLC in kumquat seeds was higher than in the peels, ranging from 24.85 ± 2.19 to 93.99 ± 5.38 mg/g and 2.24 ± 0.04 to 8.43 ± 0.38 mg/g, respectively. Notably, FOTS exhibited a remarkably higher limonin content of 93.99 ± 5.38 mg/g, surpassing the TLC found in the peels and seeds of other citrus fruits. FOTS contained four limonoids: deacetylnomilin, limonin, obacunone, and nomilin. However, (Kim, 2021) detected only limonin in the pulp of Changshou kumquat. This suggests limonoids mainly accumulate in kumquat seeds. Consequently, kumquat seeds represent an excellent source of limonoids.

In this study, a total of 161 flavonoid metabolites were identified in the peels and seeds of three kumquat varieties. The findings revealed that 35 flavonoid metabolites were up-regulated in the peels, while 18 flavonoids showed higher expression in the seeds. Notably, flavan 3-(4-Hydroxybenzoyl)epicatechin and flavonoid glycoside 6”‘-O-Sinapoylsaponarin were exclusively detected in the peel. Among these compounds, neohesperidin dihydrochalcone, a chalcone derivative, is known for its high sweetness, caries-free properties, and low energy value, making it a viable sugar substitute (Tomás-Barberán and Clifford, 2000). This study also detected six chalcones and dihydrochalcones in the peels and seeds of the three kumquat cultivars, namely neohesperidin dihydrochalcone, (R)-Kanzonol Y, 2’,4-Dihydroxy-4’,6’-dimethoxychalcone, phloretin, xanthohumol D, and xanthohumol. Moreover the peels of long-lived kumquats are easier to peel, facilitating the extraction and application of chalcone compounds found in the peels. Consequently, the long-lived kumquat exhibits promising medicinal value.

The Citrus primarily contains O-glycosylated flavonoids and flavanones (Wang et al., 2017, Wang et al., 2021), while the kumquat is characterized by C-glycosylated flavonoids (Lou and Ho, 2017). The hybrid FOT showed distinct flavonoid accumulation patterns: O-glycosylated flavonoids were upregulated in the peel, while both O- and C-glycosylated flavonoids were enriched in the seeds. These metabolic features reflect contributions from both parental genera, enhancing flavonoid diversity. Both FCSLY and FCSHP exhibited significant accumulation of methylated flavonoids and polymethoxyflavones (PMFs) in their peel and seed tissues. Our analysis identified 49 O-glycosylated flavonoids and 5 C-glycosylated flavonoids across three kumquat varieties (**Supplementary Table S3**). Only three flavanones were detected: Eriodictyol, 4’,5,8-Trihydroxyflavanone, and Naringenin, confirming relatively scarce flavanone distribution in kumquat. Notably, previous studies reported that isoflavones mainly exist in legumes or Chinese medicinal materials (e.g., soybeans) (Huang et al., 2022)and play important roles in plant defense. Fu Wang et al. identified 8 isoflavones in orange peels (Wang et al., 2019). In our study, 32 isoflavones were identified in kumquat peels and seeds, such as Coumestrin, Psoralidin, Cyclokievitone, Dihydrodaidzein, Glisoflavanone, and Rotenone (**Supplementary Table S3**). Furthermore, apart from previously reported flavonoids in kumquat (e.g., Phloretin, Diosmin, Isorhamnetin, Kaempferol, Quercetin, Luteolin, and Poncirin) (Lou et al., 2015; Lou and Ho, 2017), we detected additional flavonoids in kumquat, including Naringin (previously found in pomelos) (Xi et al., 2014), Nobiletin (in Citrus) (Cheng et al., 2024), and Diosmetin (in oranges, bergamots, and lemons) (Mandalari et al., 2006; Lin and Harnly, 2007). Notably, flavonoids rarely reported in kumquat, such as Limocitrin, Isosakuranin, Orientin, Kaempferitrin, and Hesperetin, were also identified, along with their glycosylated forms (e.g., Diosmetin 7-neohesperidoside, Hesperetin 7-neohesperidoside, Luteolin 7-glucoside, Quercetin 3-O-glucosyl-rutinoside, and Orientin 2’’-rhamnoside). Our findings expand the diversity of flavonoid components in kumquat.

Phenolic acids significantly influence the flavor profile of citrus fruits and serve as important antioxidants in the peel (Bian et al., 2022; Peng et al., 2025). Both FCSHPP and FCSHPS exhibited upregulated expression of numerous phenolic acid metabolites, which may contribute substantially to their distinct flavor characteristics. In this study, we identified 44 phenolic acids in kumquat peel and seeds (**Supplementary Table S4**), including free phenolic acids (sinapic acid, gallic acid, vanillic acid, coumaroyl tartaric acid, cryptochlorogenic acid, syringic acid, benzoic acid, and salicylic acid) along with various derivatives (1-O-Feruloylglucose, 2-O-p-Coumaroyltartronic acid, Caffeoyl C1-glucuronide, and 1-O-Galloylglycerol). Previous studies reported different phenolic acid profiles in citrus species. Chen et al, detected 7 phenolic acids (gallic acid, chlorogenic acid, protocatechuic acid, caffeic acid, p-coumaric acid, ferulic acid, and benzoic acid) in the peels of citrus, pomelo, and kumquat (Chen et al., 2021). Peng et al, identified 77 phenolic acids across five citrus varieties in both pulp and peel tissues (Peng et al., 2025). The distribution patterns of phenolic acids varied significantly depending on both cultivar types and tissue parts, which may also be attributed to growth conditions and fruit ripening stages.

Secondary metabolites play a pivotal role in influencing the quality, flavor, and medicinal properties of fruits. Currently, research on kumquat fruit secondary metabolites predominantly focuses on phenols, flavonoids, limonoids, and other compounds. High-throughput detection of secondary metabolites through non-targeted metabolomics can greatly facilitate the comprehensive development and utilization of kumquat plants.

## 5 Conclusion

In this study, a total of 1719 metabolites were identified, encompassing 15 compound categories and 27 subcategories. Specifically, FCSHP exhibited high SC content levels, and abundant peel flavonoids, rendering it nutritionally valuable and well-suited for whole fruit consumption. FCSLY possesses both edible and medicinal value. The peel of FOTS is rich in flavonoids and easy to peel, making it suitable for medicinal purposes, while the seeds of FOTS serve as a favorable source of limonoids. Therefore, this study delved into the correlation mechanisms between fruit flavor, oil gland, color variation, and the secondary metabolites of three kumquat varieties, providing valuable insights for the practical application of kumquats.

## Data Availability

The original contributions presented in the study are included in the article/**Supplementary Material**. Further inquiries can be directed to the corresponding author.

## References

[B1] Abirami A. Nagarani G. Siddhuraju P. (2014). *In vitro* antioxidant, anti-diabetic, cholinesterase and tyrosinase inhibitory potential of fresh juice from Citrus hystrix and C. maxima fruits. Food Sci. Hum. Wellness. 3 (1), 16–25. doi: 10.1016/j.fshw.2014.02.001

[B2] Aravind S. Barik D. Ragupathi P. Vignesh G. (2021). Investigation on algae oil extraction from algae Spirogyra by Soxhlet extraction method. Materials Today: Proc. 43, 308–313. doi: 10.1016/j.matpr.2020.11.668

[B3] Barreca D. Bellocco E. Caristi C. Leuzzi U. Gattuso G. (2011). Kumquat (Fortunella japonica Swingle) juice: Flavonoid distribution and antioxidant properties. Food Res. Int. 44, 2190–2197. doi: 10.1016/j.foodres.2010.11.031

[B4] Bentley M. D. Rajab M. S. Mendel M. J. Alford A. R. (1990). Limonoid model insect antifeedants. J. Agric. Food Chem. 38, 1403–1406. doi: 10.1021/jf00096a022

[B5] Bian X. Xie X. Cai J. Zhao Y. Miao W. Chen X.. (2022). Dynamic changes of phenolic acids and antioxidant activity of Citri Reticulatae Pericarpium during aging processes. Food Chem. 373, 131399. doi: 10.1016/j.foodchem.2021.131399, PMID: 34717083

[B6] Can L. Jing L. Yonghai R. Nvyong L. Long R. (2012). Aqueous extraction of limonin from Citrus reticulate Blanco. Czech J. Food Sci. 30, 364–368. doi: 10.17221/108/2011-CJFS

[B7] Chen Y. Pan H. Hao S. Pan D. Yu W. (2021). Evaluation of phenolic composition and antioxidant properties of different varieties of Chinese citrus. Food Chem. 1), 130413. doi: 10.1016/j.foodchem.2021.130413, PMID: 34175629

[B8] Cheng Y. Feng S. Sheng C. Yang C. Li Y. (2024). Nobiletin from citrus peel: a promising therapeutic agent for liver disease-pharmacological characteristics, mechanisms, and potential applications. Front. Pharmacol. 15. doi: 10.3389/fphar.2024.1354809, PMID: 38487166 PMC10938404

[B9] China National Institute Of Standardization (2020). “GB/T 39558–2020 Sensory analysis—Methodology—”A”-”not A” test,” (Beijing: State Administration for Market Regulation and the Standardization Administration of China).

[B10] Dongjiang Gong G. (2006). *Descriptors and Data Standard for Citrus (Citrus* spp.*).* (Beijing: China Agriculture Press).

[B11] Essoung F. R. E. Chhabra S. C. Mba’ning B. M. Mohamed S. A. Lwande W. Lenta B. N.. (2018). Larvicidal activities of limonoids from Turraea abyssinica (Meliaceae) on Tuta absoluta (Meyrick). J. Appl. Entomology 142, 397–405. doi: 10.1111/jen.12485

[B12] Flamini G. Tebano M. Cioni P. L. (2007). Volatiles emission patterns of different plant organs and pollen of Citrus limon. Analytica Chimica Acta 589, 120–124. doi: 10.1016/j.aca.2007.02.053, PMID: 17397661

[B13] Hu F. G. Bi X. F. Fu X. F. Li Y. A. Li G. P. Li Y. Q.. (2023). Comparative metabolome profiles and antioxidant potential of four coffea arabica L. Varieties differing in fruit color. Diversity-Basel 15, 724. doi: 10.3390/d15060724

[B14] Huang L. Zheng T. Hui H. Xie G. (2022). Soybean isoflavones modulate gut microbiota to benefit the health weight and metabolism. Front. Cell. Infection Microbiol. 12. doi: 10.3389/fcimb.2022.1004765, PMID: 36118025 PMC9478439

[B15] Jimenez A. Mata R. Pereda-Miranda R. Calderon J. Isman M. B. Nicol R.. (1997). Insecticidal Limonoids from Swietenia humilis and Cedrela salvadorensis. J. Chem. Ecol. 23, 1225–1234. doi: 10.1023/B:JOEC.0000006460.25281.9d

[B16] Kim H. J. (2021). Comparative metabolomics analysis of citrus varieties. Foods 10, 2826. doi: 10.3390/foods10112826, PMID: 34829107 PMC8622604

[B17] Li X. Meenu M. Xu B. (2023). Recent development in bioactive compounds and health benefits of kumquat fruits. Food Rev. Int. 39 (7), 4312–4332. doi: 10.1080/87559129.2021.2023818

[B18] Lin L. Z. Harnly J. M. (2007). A screening method for the identification of glycosylated flavonoids and other phenolic compounds using a standard analytical approach for all plant materials. J. Agric. Food Chem. 55, 1084. doi: 10.1021/jf062431s, PMID: 17256956 PMC3762687

[B19] Liu X. Liu B. Jiang D. Zhu S. Shen W. Yu X.. (2019). The accumulation and composition of essential oil in kumquat peel. Scientia Hortic. 252, 121–129. doi: 10.1016/j.scienta.2019.03.042

[B20] Lou S. N. Ho C. T. (2017). Phenolic compounds and biological activities of small-size citrus: Kumquat and calamondin. J. Food Drug Anal. 25, 162–175. doi: 10.1016/j.jfda.2016.10.024, PMID: 28911534 PMC9333435

[B21] Lou S. N. Lai Y. C. Huang J. D. Ho C. T. Ferng L. H. A. Chang Y. C. (2015). Drying effect on flavonoid composition and antioxidant activity of immature kumquat. Food Chem. 171, 356–363. doi: 10.1016/j.foodchem.2014.08.119, PMID: 25308680

[B22] Ma Q. (2019). *Study on the change of nutrition and functional components in Nanfeng tangerine during ripening* . (master's thesis). (Nanchang (Jiangxi): Jiangxi Science and Technology Normal University). doi: 10.27751/d.cnki.gjxkj.2019.000161

[B23] Ma Q. L. Hu Y. W. Dong X. H. Zhou G. F. Liu X. Gu Q. Q.. (2021). Metabolic profiling and gene expression analysis unveil differences in flavonoid and lipid metabolisms between ‘Huapi’ Kumquat (Fortunella crassifolia swingle) and its wild type. Front. Plant Sci. 12. doi: 10.3389/fpls.2021.759968, PMID: 34925410 PMC8675212

[B24] Mandalari G. Bennett R. N. Bisignano G. Saija A. Dugo G. Lo Curto R. B.. (2006). Characterization of flavonoids and pectins from bergamot (Citrus bergamia Risso) peel, a major byproduct of essential oil extraction. J. Agric. Food Chem. 54, 197–203. doi: 10.1021/jf051847n, PMID: 16390199

[B25] Min-Hung C. Kai-Min Y. Tzou-Chi H. Mei-Li W. (2017). Traditional small-size citrus from Taiwan: essential oils, bioactive compounds and antioxidant capacity. Medicines 4, 28. doi: 10.3390/medicines4020028, PMID: 28930243 PMC5590064

[B26] Montoya C. González L. Pulido S. Atehortúa L. Robledo S. M. (2019). Identification and quantification of limonoid aglycones content of Citrus seeds. Rev. Bras. Farmacognosia-Brazilian J. Pharmacognosy 29, 710–714. doi: 10.1016/j.bjp.2019.07.006

[B27] Ogawa K. Kawasaki A. Omura M. Yoshida T. Ikoma Y. Yano M. (2001). 3’,5’-Di-C-beta-glucopyranosylphloretin, a flavonoid characteristic of the genus Fortunella. Phytochemistry 57, 737–742. doi: 10.1016/s0031-9422(01)00132-7, PMID: 11397442

[B28] Palma A. D’Aquino S. (2018). “Kumquat— Fortunella japonica,” in Exotic Fruits. (San Diego, CA: Academic Press), 271–278.

[B29] Peng Y. Cui X. Sun M. Huang X. Tang K. Hu B.. (2025). Liquid chromatographyTandem mass spectrometry analysis of primary metabolites and phenolic acids across five citrus species. Curr. Issues Mol. Biol. 47, 223. doi: 10.3390/cimb47040223, PMID: 40699622 PMC12026233

[B30] Qingguo Tian L. D. (1999). A rapid and simple spectrophotometric method for the determination of limonoids in citrus seeds. J. Instrumental Chem. 18, 456–460. doi: 10.3969/j.issn.1004-4957.1999.05.015

[B31] Sadek E. S. Makris D. P. Kefalas P. (2009). Polyphenolic composition and antioxidant characteristics of kumquat (Fortunella margarita) peel fractions. Plant Foods Hum. Nutr. 64, 297–302. doi: 10.1007/s11130-009-0140-1, PMID: 19866359

[B32] Shunming F. Chunling Z. Ting L. Jiaqi W. Yu T. Zhimin C.. (2019). Limonin: a review of its pharmacology, toxicity, and pharmacokinetics. Molecules 24, 3679. doi: 10.3390/molecules24203679, PMID: 31614806 PMC6832453

[B33] Siau Sie V. Grimes H. D. Lange B. M. (2012). Assessing the biosynthetic capabilities of secretory glands in Citrus peel. Plant Physiol. 159, 81–94. doi: 10.1104/pp.112.194233, PMID: 22452856 PMC3375987

[B34] Sun X. S. Wang Z. Li X. Du S. H. Lin D. M. Shao Y. X. (2023). Effects of Yucca schidigera extract on serum biochemical parameters, humoral immune response, and intestinal health in young pigeons. Front. Veterinary Sci. 9. doi: 10.3389/fvets.2022.1077555, PMID: 36713856 PMC9878700

[B35] Tan S. Zhao X. Yang Y. Ke Z. Zhou Z. (2016). Chemical profiling using uplc Q-tof/ms and antioxidant activities of fortunella fruits. J. Food Sci. 81, C1646–C1653. doi: 10.1111/1750-3841.13352, PMID: 27243926

[B36] Tomás-Barberán F. A. Clifford M. N. (2000). Flavanones, chalcones and dihydrochalcones – nature, occurrence and dietary burden. J. Sci. Food Agric. 80, 1073–1080. doi: 10.1002/(SICI)1097-0010(20000515)80:73.0.CO;2-B

[B37] Wang F. Chen L. Chen H. Chen S. Liu Y. (2019). Analysis of flavonoid metabolites in citrus peels (Citrus reticulata “Dahongpao”) using UPLC-ESI-MS/MS. Molecules 24, 2680. doi: 10.3390/molecules24152680, PMID: 31344795 PMC6696472

[B38] Wang S. Yang C. Tu H. Zhou J. Liu X. Cheng Y.. (2017). Characterization and metabolic diversity of flavonoids in citrus species. Sci. Rep. 7, 10549. doi: 10.1038/s41598-017-10970-2, PMID: 28874745 PMC5585201

[B39] Wang Y. Liu X. J. Chen J. B. Cao J. P. Sun C. D. (2021). Citrus flavonoids and their antioxidant evaluation. Crit. Rev. Food Sci. Nutr. 2), 1–22. doi: 10.1080/10408398.2020.1870035, PMID: 33435726

[B40] Wu W. Jiao C. Li H. Ma Y. Liu S. (2018). LC-MS based metabolic and metabonomic studies of Panax ginseng. Phytochem. Anal. 29, 331–340. doi: 10.1002/pca.2752, PMID: 29460310

[B41] Xi W. Fang B. Zhao Q. Jiao B. Zhou Z. (2014). Flavonoid composition and antioxidant activities of Chinese local pummelo (Citrus grandis Osbeck.) varieties - ScienceDirect. Food Chem. 161, 230–238. doi: 10.1016/j.foodchem.2014.04.001, PMID: 24837945

[B42] Xu J. Deng X. X. (2002). Red juice sac of citrus and its main pigments. Int. J. Fruit Sci. 5, 30–36. doi: 10.3969/j.issn.1009-9980.2002.05.006

[B43] Yasuda K. Yahata M. Kunitake H. (2016). Phylogeny and classification of kumquats (Fortunella spp.) inferred from CMA karyotype composition. Hort J. 85, 115–121. doi: 10.2503/hortj.MI-078

[B44] Ze Y. Shuai J. Yuduan D. Zhuang W. Huijun G. Zhiyong P.. (2012). Comparative transcriptomics and proteomics analysis of citrus fruit, to improve understanding of the effect of low temperature on maintaining fruit quality during lengthy post-harvest storage. J. Exp. Bot. 63, 2873–2893. doi: 10.1093/jxb/err390, PMID: 22323274 PMC3350911

[B45] Zhang Y. (2019). Analysis of the distribution status and climate suitable distribution areas of Fortunella in China. (dissertation/master's thesis). (Hunan Agricultural University). doi: 10.27136/d.cnki.ghunu.2019.000525

